# Quantitation of L-cystine in Food Supplements and Additives Using ^1^H qNMR: Method Development and Application

**DOI:** 10.3390/foods12122421

**Published:** 2023-06-20

**Authors:** Zhen Chen, Xiaofang Lian, Meichen Zhou, Xiuli Zhang, Cong Wang

**Affiliations:** 1 School of Pharmacy, Jiangsu University, Zhenjiang 212013, China; chenzhen@ujs.edu.cn; 2 Key Laboratory of Marine Drugs, Ministry of Education, School of Medicine and Pharmacy, Ocean University of China, Qingdao 266003, China; xiaofanglian0365@163.com (X.L.); 21200811129@stu.ouc.edu.cn (M.Z.); xiulizhang@ouc.edu.cn (X.Z.); 3 Institute of Medicinal Biotechnology, Chinese Academy of Medical Sciences & Peking Union Medical College, Beijing 100050, China; 4 Laboratory for Marine Drugs and Bioproducts of Qingdao Pilot National Laboratory for Marine Science and Technology, Qingdao 266237, China

**Keywords:** L-cystine, food supplements, food additives, quantitative NMR, food analysis, food quality assessment, method optimization, method development and validation

## Abstract

Cystine-enriched food supplements are increasingly popular due to their beneficial health effects. However, the lack of industry standards and market regulations resulted in quality issues with cystine food products, including cases of food adulteration and fraud. This study established a reliable and practical method for determining cystine in food supplements and additives using quantitative NMR (qNMR). With the optimized testing solvent, acquisition time, and relaxation delay, the method exhibited higher sensitivity, precision, and reproducibility than the conventional titrimetric method. Additionally, it was more straightforward and more economical than HPLC and LC-MS. Furthermore, the current qNMR method was applied to investigate different food supplements and additives regarding cystine quantity. As a result, four of eight food supplement samples were found to be inaccurately labeled or even with fake labeling, with the relative actual amount of cystine ranging from 0.3% to 107.2%. In comparison, all three food additive samples exhibited satisfactory quality (the relative actual amount of cystine: 97.0–99.9%). Notably, there was no obvious correlation between the quantifiable properties (price and labeled cystine amount) of the tested food supplement samples and their relative actual amount of cystine. The newly developed qNMR-based approach and the subsequent findings might help standardization and regulation of the cystine supplement market.

## 1. Introduction

L-cystine is a sulfur-containing amino acid found in dietary sources such as eggs, meats, dairy products, and whole grains [1]. L-cystine, together with L-cysteine, is produced through the enzymatic conversion from 2-amino-Δ2-thiazoline-4-carboxylic acid by microorganisms, and cystine can be formed via oxidation of cysteine in normoxic conditions [2,3,4]. As a nutritional food additive, cystine, being a crucial component of α-keratin, has also been recognized as a safe and beneficial food component since it plays a fundamental role in maintaining the growth of healthy skin, hair, nails, and bones [5,6]. Moreover, cystine serves as an essential precursor for the endogenous synthesis of an endogenous antioxidant, glutathione, which prevents free radical damage and supports liver detoxification [7,8,9]. Therefore, despite the fact that it is a non-essential amino acid, supplementation of cystine has been of increased interest in recent years.

Currently, cystine-containing food products and nutraceuticals are available on the market, for most of which the main benefits include stimulating collagen formation, improving skin elasticity, accelerating skin healing in burns and wounds, and preventing hair loss [10]. Some of the cystine-based functional supplements are also claimed to protect the liver and brain, alleviate alcohol-induced damage, reduce the effects of hangovers [11], and even prevent disorders related to oxidative stress, such as atherosclerosis and cancers [12,13]. On the other side, too much cystine in the body can lead to the condition cystinuria, which is a common cause of bladder stones or kidney stones. Thus, cystine serves as a beneficial food component; controlling and monitoring its content is of great importance.

In the field of food supplements, it is crucial to disclose clear and accurate information regarding the functional ingredients. It is known that there are considerable food quality and food adulteration/fraud issues with some food supplements commercially available, such as misleading promotion of the functions, including inaccurate content of the functional ingredient(s), or even incorrect labeling of them. Therefore, precise, practical, and reproducible standards should be established for controlling the quality and ensuring the safety of these food supplements. It is imperative that national and community regulations provide adequate measures to ensure the quality and safety of supplements that are made available to consumers. For cystine, there is currently no established regulation for determining its content in dietary supplements in China. While the *Chinese Pharmacopoeia* (ChP, 2020 Edition) prescribes the titration method for measuring its content [14], which is susceptible to environmental influence, prone to subjective errors or mistakes, and laborious, rendering it unsuitable for high-throughput applications. In recent years, determination using an amino acid analyzer has been increasingly employed for cystine quantification in food products [15], as well as high-performance liquid chromatography (HPLC) and liquid chromatography-mass spectrometry (LC-MS) with or without derivatization [16]. These techniques exhibit exceptional specificity with the help of chromatographic separation. In comparison, our interest is to directly assess L-cystine in cystine-enriched food as well as food supplements and food additive samples in pursuit of a more economical and easier-to-operate approach.

Quantitative nuclear magnetic resonance spectroscopy (qNMR) has been applied to food analysis, such as qualitative and quantitative analyses of food components, food metabolomics, and food resource exploration [17,18,19], as it enables a more practical, precise, and accurate analysis with more straightforward sample preparation (without separation or derivation). Yet, to the best of our knowledge, there has not been a report on L-cystine determination in food-related samples. Therefore, in the present study, a fast and accurate quantitative method was developed for cystine in food supplements and food additives using ^1^H NMR. The optimization of the sample pretreatment and instrumental parameters, the method validation, and its application to different commercially available products were conducted and discussed.

## 2. Materials and Methods

### 2.1. Chemicals

The deuterated solvents used for ^1^H NMR tests, including deuterium oxide (D_2_O, CAS No. 7789-20-0, >99.5%), deuterium chloride (DCl, CAS No. 7698-05-7, >99.9%), and sodium deuteroxide (NaOD, CAS No. 14014-06-3, >99.5%) were purchased from Cambridge Isotope Laboratories Inc. (Andover, MA, USA). The reference standard L-cystine (CAS No. 56-89-3, ≥98%) and the internal standard (ISTD) maleic acid (CAS No. 110-16-7, ≥99%) were obtained from Sigma-Aldrich (Saint Louis, MO, USA). ISTD was diluted to 60 µL/mL using either D_2_O, DCl, or NaOD before use. Unless otherwise specified, other reagents and solvents were of analytical grade, which were purchased from Shandong Yuwang Industrial Co., Ltd. (Yucheng, China).

### 2.2. Sample Collection and Pretreatment for qNMR Test

Cystine-enriched supplement samples were purchased from local markets and online, and the detailed sample information is listed in Table 1.

For tablets, the obtained samples were grounded to fine powder; for capsules, the shells of the samples were removed and the powder inside was collected; for oral liquids, the liquid was aspirated; and for softgels, the soft shells were pricked and the oil inside was collected. Next, 20 mg of the pretreated sample was taken into a 2 mL Eppendorf^®^ Safe-Lock tube and weighed precisely on an ultrasensitive electro-balance (BCE224-1CCN, Sartorius Inc., Göttingen, Germany). Thereafter, the sample was added with 1000 µL of the deuterated solvent (D_2_O, DCl, or NaOD, containing 0.06 mg/mL of ISTD; the favorable solvent was based on the comparison result below in Section 3.1). Then, the sample was extracted using ultrasonication for 15 min (KQ-250E, Kunshan Ultrasonic Instruments Co., Ltd., Suzhou, China), followed by centrifugation at 13,000 rpm for 3 min (D3024, DLAB Scientific Inc., Beijing, China) to remove the residue. Finally, 600 uL of the supernatant was carefully aspirated for the qNMR test. For each tested sample, the analysis was conducted in quadruplicates.

### 2.3. Quantitation of Cystine by Conventional Titrimetric Analysis

**Scheme 1:**
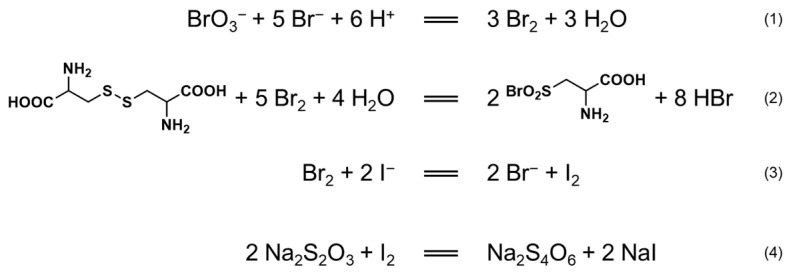
Reactions in titration method for cystine measurement. (**1**) Preparation of Br_2_ in the acidic environment, (**2**) cystine reacted with Br^2^ in the acidic environment, (**3**) depletion of excess Br_2_ with KI, (**4**) titration of I_2_ with Na_2_S_2_O_3_ solution and monitoring the endpoint with starch indicator.

The analytical procedure was accorded to ChP 2020 Edition [14]. Briefly, 80 mg of the sample was dissolved in 12 mL of NaOH solution, then placed in the ice bath in a dark room for 10 min with the addition of 10 mL of KBr solution, 50 mL KBrO_3_ solution, and 15 mL HCl solution. Next, the sample was added with 1.5 g of KI and shaken for 1 min, followed by titration with Na_2_S_2_O_3_. When approaching the titration end (the color of the solution started to change from brownish red to pale green), 2 mL of the starch indicator was added (the color changed to blue). The titration continued until the blue color just disappeared. The reactions in the whole procedure are shown in Scheme 1 below:

### 2.4. ^1^H NMR Analysis

#### 2.4.1. Apparatus

^1^H NMR spectra were recorded on an Agilent 500-DD2 spectrometer (500 MHz, Agilent Technologies, Inc., Santa Clara, CA, USA) equipped with a 5 mm OneNMR Probe and a 7510-AS sample changer. Data collection was carried out under a controlled temperature of 25 °C and spectral width of 8012.8 Hz. The pulse angle, the number of scans, and line broadening, which are associated with signal-to-noise-ratio, were set as 45°, 32 times, and 0.3 Hz, respectively.

#### 2.4.2. Optimization of qNMR Conditions

For qNMR experiments, acquisition time and relaxation delay, as two important parameters affecting signal intensities, are involved in the precise and complete acquirement of the NMR peaks [20], and, thus, need to be optimized in detail. The length of acquisition time affects the resolution of the spectrum, of which a proper value contributes to flattening the baseline and improving the resolution and is commonly set within the range of 2–4 s [20]. Therefore, the acquisition time from 1 s to 4 s was optimized in the present work. While the length of relaxation delay, referring to the equilibrium establishment between pulses, impacts the integrity of signals [21,22]. Here, in this experiment, a series of relaxation delay times from 1 s to 25 s were comparatively investigated.

#### 2.4.3. NMR Data Processing

MestReNova 9.0 (Mestrelab Research, S.L., Santiago de Compostela, Spain) was used to process all the acquired raw data, including phase correction, baseline adjustment, and integration, which were conducted manually, as previously described [18,23]. The average values from six times replicated processing were performed for all the phase correction and integration procedures to minimize analyst error [24]. The chemical shifts were referenced to the IS resonance (*δ* 5.86, s), and the obtained peak area was used for the quantitation. The calculation of the analytes was accorded to our previous work [18] using the formula below:

(1) $$W_{s}=W_{r}\times \frac{A_{s}}{A_{r}}\times \frac{E_{s}}{E_{r}}$$

where W_s_ stands for the weight of IS, A_s_ and A_r_ denote the peak area of analyte and ISTD, respectively, while E_s_ and E_r_ represent the ratios of molecular weight to nuclei number for analyte and IS, respectively.

### 2.5. Validation of the qNMR Method

The developed method was validated according to the guidelines addressed by ChP 2020 Edition [14]. Specificity was confirmed by comparing the blank (background), the cystine standard (positive control), and the food supplement sample without cystine (negative control). The calibration curve was drawn using a series of diluted cystine solutions (D_2_O, DCl, or NaOD; the favorable solvent was based on the comparison result below in Section 3.1) with the concentration of 0.1, 0.5, 1.0, 5.0, 10.0, and 20.0 mg/mL (with 0.06 mg/mL of ISTD in each sample), and the linear regression was implemented by plotting the peak area ratio of cystine and the IS (A_s_/A_r_, y-axis) vs. cystine concentration (x-axis, in mg/mL). Sensitivity was evaluated as the limit of detection (LOD) and the limit of quantification (LOQ), which were defined at a signal-to-noise ratio (S/N) of 3 and 10, respectively. For intra-assay precision (repeatability) and intermediate precision (reproducibility), six replicates (*n* = 6) of the independently prepared samples were assessed. Stability was evaluated by repeatedly testing the same sample at the time of 0, 2, 4, 12, 24, 48, and 72 h (*n* = 3 for each time). Accuracy, reflected by recovery tests, was determined at three spiking levels (50%, 100%, and 200% of the original level, *n* = 6 for each) and calculated with the formula below:

(2) $$Recovery\left(\%\right)=\frac{{Amount}_{found}-{Amount}_{original}}{{Amount}_{spiked}}\times 100\%$$

### 2.6. Data Analysis and Statistics

The obtained data are presented as means ± standard deviation (SD). One-way ANOVA with Tukey’s post hoc test was conducted, and *p* < 0.05 was considered to be statistically significant. Correlation analysis was performed using Prism 9.4.0 (GraphPad Software, Inc., San Diego, CA, USA), and the Pearson correlation coefficient was calculated in this study.

## 3. Results and Discussion

### 3.1. Detection of Cystine Using ^1^H NMR and Selection of the Deuterated Solvents

**Figure 1:**
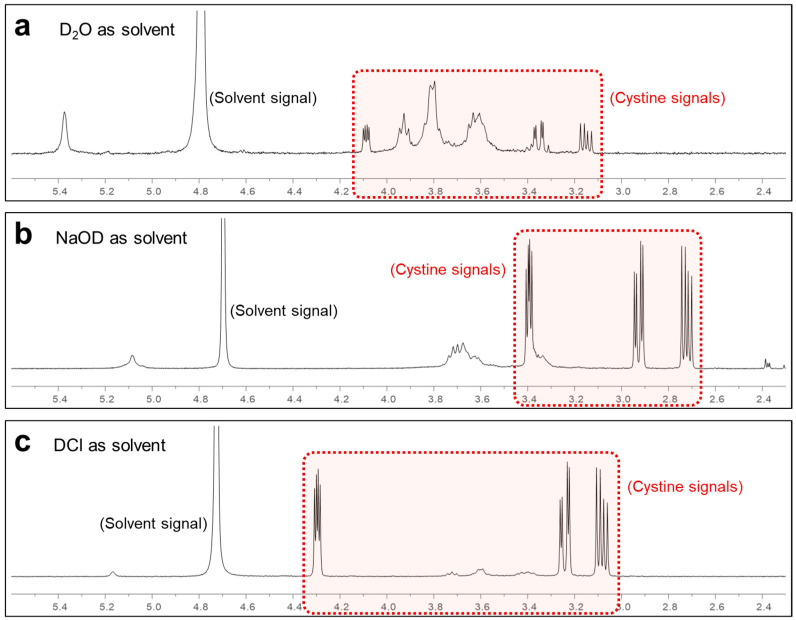
^1^H NMR spectrum of cystine sample with ISTD in D_2_O (**a**), NaOD (**b**), and DCl (**c**).

Concerning the solubility of cystine, we initially compared the suitability of three solutions (i.e., D_2_O, DCl, and NaOD) for this experiment. The favorable solvents should be able to dissolve the analyte completely, and there should be no overlap of signals in the obtained spectra. The representative ^1^H NMR spectra of the cystine-enriched sample with D_2_O, DCl, and NaOD are shown in Figure 1. As a result, NaOD showed the best solubility and the least interference among the three deuterated solvents, which can prevent the error caused by the partial dissolution of the sample. Moreover, the spectra obtained using NaOD showed the sharpest peak shapes within the most concentrated chemical shift regions for all signals, while those using D_2_O resulted in the most interference, making it most difficult to recognize the signals. Based on these results, the suitable solvent for the following experiments in this study was decided to be NaOD, including method development and application.

**Figure 2:**
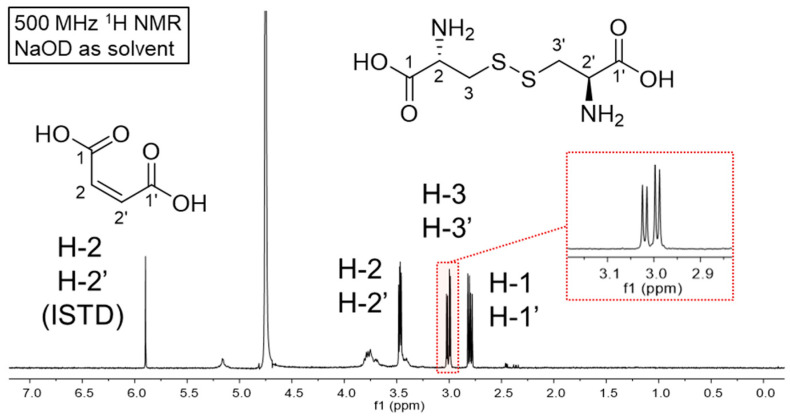
Assignment of cystine together with ISTD (maleic acid) in the representative ^1^H NMR spectrum of the food supplement sample (NaOD as solvent).

The typical ^1^H NMR spectrum of the cystine-enriched food supplement sample with ISTD using NaOD as solvent is shown in Figure 2, in which δ 3.49 (dd, *J* = 7.6, 4.7 Hz, H-2 and H-2’), δ 3.02 (dd, *J* = 13.7, 4.7 Hz, H-3a and H-3’a), and δ 2.81 (dd, *J* = 13.7, 7.6 Hz, H-3b and H-3’b) provided informative structural features of cystine, while δ 5.86 (s) conducted the resonance of ISTD (H-2 and -3 of maleic acid). The solvent peak of NaOD was located at δ 4.75 ppm, which did not interfere with the signals of the analyte or the ISTD. Subsequently, the combined signals of H-3a and H-3’a (δ 3.02, dd, *J* = 13.7, 4.7 Hz) were selected as the quantitation reference peaks, as they were identical and highly specific.

### 3.2. Optimization of qNMR Parameters

#### 3.2.1. Acquisition Time Length

As acquisition time is closely related to the resolution of the obtained spectrum, firstly, 1, 1.5, 2, 2.5, 3, 3.5, and 4 s as the length of the acquisition time were compared for evaluating whether it could comply with the time of the signal completely decaying into noise [20]. An excessively long acquisition time results in more noise and leads to a decrease in the spectrum resolution, and insufficient acquisition time may truncate the free-sensing decay signal, resulting in false signals. In this study, the results showed that acquisition for 2 s enabled the FID signals to be attenuated entirely to meet the requirements of quantification, which was, therefore, set as the acquisition time in the following experiments.

#### 3.2.2. Relaxation Delay Length

**Figure 3:**
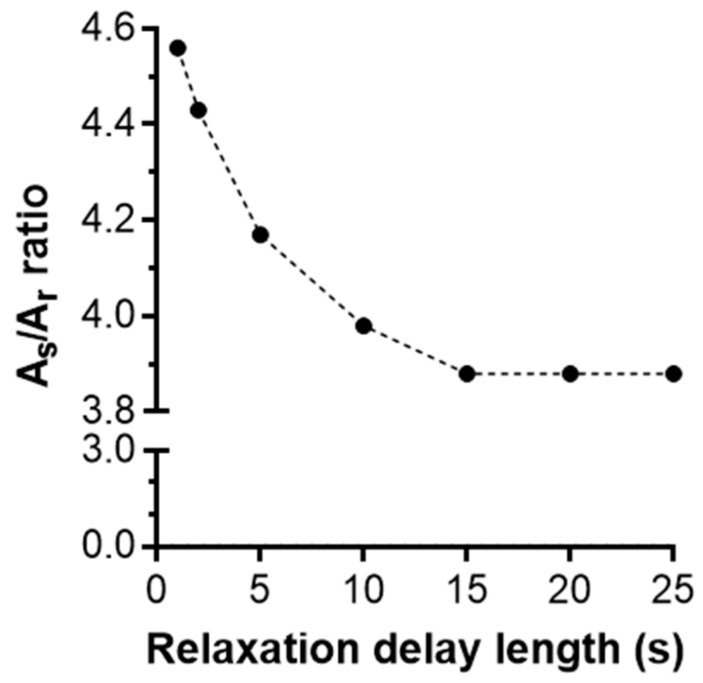
Effect of the relaxation delay length and the A_s_/A_r_ ratio of cystine.

The length of the relaxation delay was also investigated. The times of 1, 2, 5, 10, 15, 20, and 25 s were selected, and for each value, the obtained A_s_/A_r_ ratio was calculated (Figure 3). The archived curve showed that from 1 to 10 s, the A_s_/A_r_ ratio sharply decreased, indicating an incomplete gain of the signals, and after 15 s the values tended to be stable. For ensuring that the relaxation delay was sufficient and not exceeded, the length of 20 s was set as the final value in the following experiments.

### 3.3. Validation of the Developed Method

**Figure 4:**
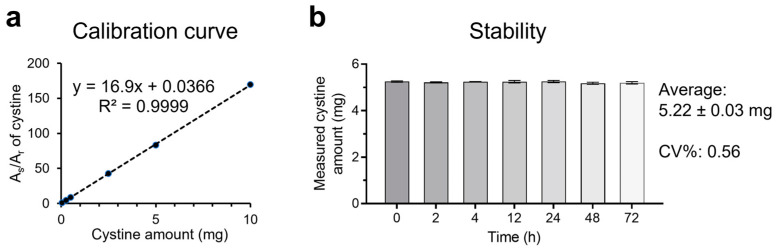
(**a**) Calibration curve of cystine and (**b**) stability assay for the developed method.

Subsequently, the feasibility and validity of the method were confirmed under the optimized conditions. The calibration curve of cystine showed a satisfied linearity (R^2^ = 0.9999) of this method within the linear range (from 0.05 mg to 10 mg) (Figure 4a). The LOD and LOQ were 3.8 μg and 12.5 μg, respectively, suggesting sufficient sensitivity for the current purpose (Table 2). The intra-assay and intermediate precision also fit the requirements of ChP, with CV values of 0.93% and 2.20%, respectively (Table 2). For accuracy, the recovery rates of cystine in all three spiking levels were within the range of 100.7–103.3% (Table 2). In terms of stability, there was no significant variation (*p* > 0.05 for all) in the sample for 72 h after preparation (Figure 4b). Therefore, the currently developed quantitative method using ^1^H NMR was validated and feasible for determining cystine in food supplements. Moreover, it is more sensitive, precise, and reproducible than the conventional titrimetric method (Table 2).

Although there have recently been reports of cystine determination applied to food sciences using HPLC or LC/MS with or without derivatization, the current method based on ^1^H NMR expressed comparable repeatability and reproducibility, as well as sufficient linearity and sensitivity. Moreover, the proposed qNMR method without separation simplified sample pretreatment and shortened the instrument running time compared to chromatographic approaches: the current qNMR method required 11 min for testing one sample, compared to 20 min for LC-MS [16] and more than 30 min for HPLC-based approaches [15,25,26,27], as reported in the literature. Therefore, the currently developed ^1^H NMR-based quantitative method was considered more suitable for high-throughput analysis in food sciences.

### 3.4. Quantitation of Cystine in Food Supplements

#### 3.4.1. Variations of Cystine Amount

With the established method, the amount of cystine was determined in the 8 kinds of food supplements and 3 food additives (*n* = 4 for each sample, listed in Table S1), and the resulting data are shown in Table 3. The relative actual amount of cystine was calculated as the ratio of the measured amount to the labeled amount for the same sample. For food supplements, despite the fact that different products claimed distinguishing cystine amounts (ranging from 8.0 to 60.0 mg per serving), the results revealed quite distinctive values of relative amount: 5 of 8 samples showed highly qualified results (from 96.6% ± 2.5% to 101.4% ± 2.1%, within the range of ±5% of the labeled values). The No. 7 sample was found to be even a slightly higher amount than labeled (107.2% ± 3.3%), while the other three samples (No. 3, No. 4, and No. 8) showed significantly lower amounts than labeled, indicating the occurrence of food adulteration; in particular, the No. 8 sample was found to contain almost no cystine, indicating a food fraud case. In terms of food additive samples, all three showed satisfactory quality, with the relative actual amount from 97.0% ± 1.8% to 99.9% ± 1.1%, despite their price being considerably varied (USD 17.37–50.00 per kg). Therefore, it was thought that the quality control of cystine in food additives was more standardized and reassuring compared to food supplements. 

#### 3.4.2. Correlations among the Properties of Different Food Supplements and Their Relative Actual Amount of Cystine

In order to explore the underlying factors that affect the quality of these food supplements regarding the relative amount of cystine, we investigated the connection between the quantifiable characteristics of the tested samples and their relative actual amount of cystine. As one of the most important factors for consumers when selecting health supplements, the effect of price was first investigated (Figure S1a). The samples in this experiment were separated into two price ranges: USD 0.2–0.6 per serving and USD 1.40–1.8 per serving. It was considered that for those more expensive supplements, the quality control of the active ingredient (i.e., cystine in this study, other components were not investigated) should be more rigorous and its content should comply with the labeling. As a result, in each range, there were both products with satisfied quality control (relative cystine content reached or close to 100%) and those with quality issues, of which the relative cystine content was obviously inadequate. Significantly, the No. 8 sample exhibited an extremely low concentration of cystine upon testing, despite its comparatively high price among all the samples (USD 1.44 per serving). These results indicated that price was not a decisive factor in the relative actual content of cystine (*r* = −0.15488, *p* = 0.7072), which was consistent with literature that investigated the content-related quality of nutraceuticals in food supplements [28,29]. Moreover, the relationship between the labeled cystine content per serving and its relative actual content for these products was analyzed (Figure S1b). Some samples with lower labeled cystine content (No. 1, No. 2, No. 5, and No. 7; no more than 12.5 mg per serving) showed acceptable levels (96.6–107.2%), while the sample with the highest labeled content (No. 6) also showed adequate content (98.6% ± 1.0%). However, no specific pattern was found between the variations of labeled content and relative actual content, nor significant correlation (*r* = −0.0390, *p* = 0.9263). These results suggested that the cystine supplement market might lack regulation and industry standards; unethical practices by unethical food supplement providers were currently in a stage of rampant growth and challenging to monitor or predict.

It has been recognized that standard and reliable analytical methods for functional ingredients can contribute to the development of the food products, such as the polyunsaturated fatty acids in fish oil [30], isoflavones in soybeans [31], and polyphenols in grape pomace [32]. Therefore, establishing the regulatory standards using appropriate methods and ensuring product access availability for consumers can enhance market transparency and inspire the development of the industry. In terms of cystine, it might be considered that the newly developed approach may help promote standardization and regulation for cystine supplement producers, thereby encouraging the healthy development of this field.

### 3.5. Study Limitations

It should be noted that although the developed qNMR method was straightforward, the availability and accessibility of NMR apparatus, as well as the instrumental operation and data interpretation, need considerable financial costs and qualified specialists or technicians, which made the whole approach not simple. Moreover, the present study could not fully reflect the entire market of cystine food supplements. One of the most concerning limitations was that we were unable to achieve a wider variety of samples, and therefore, the current findings were only applicable to the collected samples (now 8 food supplements; it would be more convincing if the sample number reaches 50 or more). In addition, the tested cystine food supplements expressed substantial variations in forms (tablets, capsules, oral liquids, and softgels), each with distinct functionalities and varying levels of other functional ingredients. Hence, the application of this method was also challenging due to the variations in sample matrices. In addition, although qNMR exhibited advantages over the conventional titrimetric method regarding higher sensitivity, precision, and reproducibility, complementary approaches, not only titrimetry but also LC-MS and GC-MS, could be employed to strengthen the persuasiveness of the results and gain a more comprehensive view. Furthermore, the current work only found the problems related to cystine content in food supplements without addressing wide-ranging quality control issues of other functional components contained in products, especially for those multi-component supplements that are popular in the market. Therefore, more comprehensive approaches, for instance, incorporating food fingerprinting (foodomic) techniques [33], are needed to enable a global view of the quality management of cystine food supplements.

## 4. Conclusions

In this study, a ^1^H qNMR method for determining cystine in food supplements and additives was established, together with optimization of the conditions and validation of the methodology. The proposed qNMR method was more sensitive, precise, and reproducible than the conventional titrimetric method officially used, and at the same time, more straightforward, more timesaving, and more economical than the HPLC- and the LC-MS-based approaches. Moreover, the current method was applied to eight kinds of food supplements and three kinds of food additives enriched in cystine to investigate whether there were food quality or fraud problems. As a result, half of the food supplements were revealed to be inaccurate (or even fake) in their labeling, while all the food additives exhibited qualified control of cystine. The newly developed method might help to prevent quality control issues and avoid the fraud risks of food products concerning cystine and contribute to the regulation of the amino acid food industry. In the future, a larger sample of food supplements will be tested to obtain more comprehensive information on the current status and regulatory development of the cysteine supplements market.

## Figures and Tables

**Table 1:** Information on the cystine food supplement and additive samples.

Sample Code		Functions Claimed on Product Label ^a^	Form	Labeled Cystine Amount or Purity ^b^	Other Major Functional Ingredients ^a^	Price ^c^
Food supplement						
	1	Preventing hair loss	Tablet	10.2	Vitamin B1, Vitamin B6, Vitamin B12, biotin	1.67
	2	Preventing hair loss, anti-aging	Tablet	12.5	Vitamin C, anthocyanidin extracted from blueberry	1.78
	3	Preventing hair loss, promoting hair growth	Oral liquid	25.0	Vitamin A, Vitamin B6, biotin	0.39
	4	Preventing hair loss, assisting chronic hepatitis treatment	Softgel	50.0	Vitamin B6, fish oil	1.80
	5	Relieving hangovers, protecting liver and brain from alcohol	Capsule	10.0	Vitamin B1, yeast extracts	0.24
	6	Accelerating skin regeneration	Tablet	60.0	Vitamin B2, Vitamin B6, Vitamin C, Vitamin E	0.12
	7	Preventing hair loss, preventing aging	Capsule	8.0	Crude polysaccharides extracted from radix astragali	0.63
	8	Improving skin elasticity, preventing skin aging	Softgel	15.8	Polyunsaturated fatty acids, collagen	1.44
Food additive						
	9	Enhancing dough strength	Powder	98%	N/A	18.82
	10	Enhancing dough strength	Powder	98%	N/A	17.37
	11	Enhancing dough strength	Powder	99%	N/A	50.00

^a^: Available for food supplements only. ^b^: Amount (mg per serving) for food supplements, purity (%) for food additives. ^c^: Calculated as per serving for food supplements and per kg for food additives, estimated in USD. N/A: not available.

**Table 2:** Sensitivity, intra-assay precision, intermediate precision, and accuracy of the two quantitation methods for cystine: the qNMR method and the titrimetric method.

Validation Characteristics		qNMR Method	Titrimetric Method
Sensitivity	LOD	3.8 μg	42.1 μg
	LOQ	12.5 μg	106.0 μg
Precision	Intra-assay precision (CV%)	0.93	3.59
	Intermediate precision (CV%)	2.20	4.87
Accuracy (recovery)	At 50% spiking level	103.3% ± 3.1% (CV% = 2.97)	110.3% ± 9.1% (CV% = 8.28)
	At 100% spiking level	102.1% ± 2.4% (CV% = 2.39)	107.7% ± 3.5% (CV% = 3.27)
	At 200% spiking level	100.7% ± 2.0% (CV% = 1.94)	102.0% ± 5.4% (CV% = 5.33)

**Table 3:** Cystine amounts measured in different food supplement and additive samples (*n* = 4).

Sample Code	Sample Kind	Cystine Amount (per Serving for Food Supplements, per 20 mg for Food Additives)		
		Labeled Value (mg)	Measured Value (mg)	Relative Actual Amount
1	Food supplement	10.2	9.9 ± 0.3	96.6% ± 2.5%
2	Food supplement	12.5	12.7 ± 0.3	101.4% ± 2.1%
3	Food supplement	25.0	9.3 ± 0.2	37.1% ± 0.9%
4	Food supplement	50.0	33.9 ± 2.7	67.9% ± 5.5%
5	Food supplement	10.0	9.9 ± 0.4	99.0% ± 3.6%
6	Food supplement	60.0	59.2 ± 0.6	98.6% ± 1.0%
7	Food supplement	8.0	8.6 ± 0.3	107.2% ± 3.3%
8	Food supplement	15.8	0.1 ± 0.1	0.3% ± 0.4%
9	Food additive	20.0	19.9 ± 0.4	99.4% ± 1.9%
10	Food additive	20.0	19.4 ± 0.4	97.0% ± 1.8%
11	Food additive	20.0	20.0 ± 0.2	99.9% ± 1.1%

## Data Availability

All data are contained in the article or Supplementary Materials.

## Supplementary Materials

The following supporting information can be downloaded at: https://www.mdpi.com/article/10.3390/foods12122421/s1, Table S1: cystine amount in food supplement and food additive samples; Figure S1: correlations between the relative cystine amount of food supplements and their properties.
