# Supplementary material for: Quantitation of L-cystine in Food Supplements and Additives Using 1H qNMR: Method Development and Application
Source: Foods. 2023 Jun 20;12(12):2421. doi: 10.3390/foods12122421 (PMC10297691; doi:10.3390/foods12122421)
Supplement: Supplementary file 1 [file foods-12-02421-s001.zip › foods-2438767-supplementary.pdf]

Quantitation of L-Cystine in Food Supplements and Additives Using 1H qNMR: Method Development and Application

Supporting Information

Table S1. Cystine amount in food supplement and food additive samples

Sample code	Sample kind	Labeled cystine amount (mg per serving) or purity	Measured cystine amount (per serving for food supplements, per 20 mg for food additives)					Measured cystine amount (per serving for food supplements, per 20 mg for food additives)				
			1st	2nd	3rd	4th	Average	1st	2nd	3rd	4th	Average
1	Food supplement	10.2	9.9	9.9	9.5	10.1	9.9±0.3	97.1%	97.1%	93.1%	99.0%	96.6%±2.5%
2	Food supplement	12.5	12.3	12.3	12.7	12.8	12.7±0.3	98.4%	103.2%	101.6%	102.4%	101.4%±2.1%
3	Food supplement	25.0	9.6	9.6	9.1	9.3	9.3±0.2	38.4%	36.4%	36.4%	37.2%	37.1%±0.9%
4	Food supplement	50.0	30.3	30.3	36.7	33.6	33.9±2.7	60.6%	70.2%	73.4%	67.2%	67.9%±5.5%
5	Food supplement	10.0	9.9	9.9	9.7	9.6	9.9±0.4	99.0%	104.0%	97.0%	96.0%	99.0%±3.6%
6	Food supplement	60.0	58.5	58.5	59.2	59.0	59.2±0.6	97.5%	100.0%	98.7%	98.3%	98.6%±1.0%
7	Food supplement	8.0	8.6	8.6	8.8	8.7	8.6±0.3	107.5%	102.5%	110.0%	108.8%	107.2%±3.3%
8	Food supplement	15.8	0.1	0.1	0.1	0.0	0.1±0.1	0.6%	0.0%	0.6%	0.0%	0.3%±0.4%
9	Food additive	98%	20.4	20.4	19.5	19.7	19.9±0.4	102.0%	99.5%	97.5%	98.5%	99.4%±1.9%
10	Food additive	98%	19.6	19.6	19.8	19.1	19.4±0.4	98.0%	95.5%	99.0%	95.5%	97.0%±1.8%
11	Food additive	99%	19.8	19.8	19.9	19.9	20.0±0.2	99.0%	101.5%	99.5%	99.5%	99.9%±1.1%

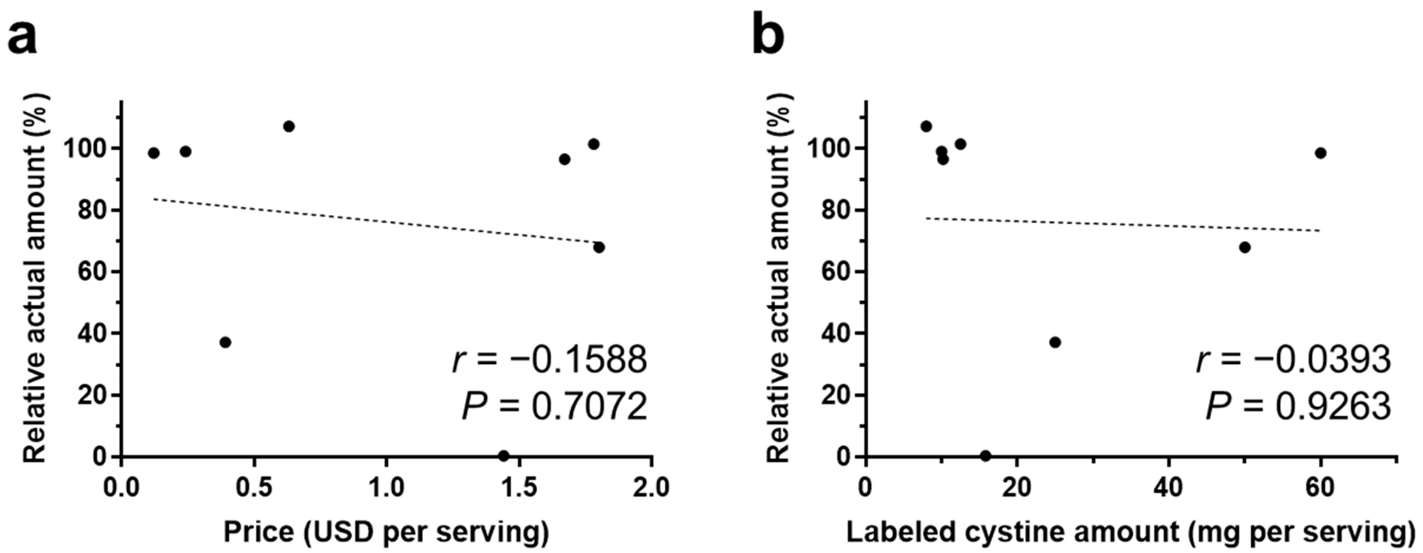

Figure S1. Correlations between the relative cystine amount of food supplements and their properties

(**a**) the price of the supplement per serving; (**b**) the labeled cystine amount per serving.
